# A Comparative Study of Colour Effects on Cognitive Performance in Real-World and VR Environments

**DOI:** 10.3390/brainsci12010031

**Published:** 2021-12-28

**Authors:** Guobin Xia, Philip Henry, Muzi Li, Francisco Queiroz, Stephen Westland, Luwen Yu

**Affiliations:** 1 School of Textile and Design, Heriot-Watt University, Scottish Borders Campus, Galashiels TD1 3HF, UK; g.xia@hw.ac.uk; 2 School of Design, University of Leeds, Woodhouse, Leeds LS2 9JT, UK; P.M.Henry@leeds.ac.uk (P.H.); f.queiroz@leeds.ac.uk (F.Q.); S.Westland@leeds.ac.uk (S.W.); 3 School of Media, Harbin Normal University, Harbin 150025, China; woodlee.ca@gmail.com

**Keywords:** colour, arousal, impulsiveness, cognitive performance, virtual reality

## Abstract

This research explores the influence of colour on cognitive performance and intellectual abilities (i.e., logical and lateral thinking abilities and people’s attention to detail) in a conventional laboratory setting and an approximately identical virtual reality (VR) environment. Comparative experiments using psychological methods were carried out in both settings to explore the impact of immersive colour experience. This work builds on earlier studies that suggest that the VR environment enhances user experiences, with results evidencing that a considered approach to colour design can trigger a positive impact on user engagement. The experiments further evaluated the positive effects of immersive colour stimuli in VR by evaluating participants’ logical and lateral thinking abilities, as well as their attention to detail. Their response time and error rate when completing each psychometric test were recorded with different hue backgrounds in both environments. The data collected from participants reveal the differential impacts of colour between the reality setting using standard colour imaging displays and in an approximately identical VR environment. Analysis of the psychometric tests shows the differential influence of colours on logical and lateral thinking abilities and people’s attention to detail between the physical environment and the VR environment. Our findings add to the data demonstrating that a well-designed immersive colour experience in VR can trigger positive user engagement and, as explored in this study, improve cognitive performance. This again positions immersive colour experience as an important design tool to be fully considered in the creation of effective VR research and applications.

## 1. Introduction

It is recognised that the success of immersive VR environments is due in part to their design and the effective trigger of human emotions and behaviours. In line with previous observations, the arguments highlight that immersion and presence are key features of the VR experience, but there are questions as to how well this experience is fully understood or whether there are greater possibilities to explore in its design potential [1,2,3,4]. Colour is recognised as a ubiquitous visual design tool that is used with great effect beyond aesthetic considerations, with about 70–80% of visual information gained through colour [5]. Colour research has demonstrated the real-world design potential of colour and light on people’s arousal and impulsiveness, affecting their emotions and cognitive performance [6,7,8]. Reviewing existing literature helps establish three pillars of VR systems: immersion, presence, and interactivity [9,10]. However, whether people’s cognitive responses to colour stimulations could be better triggered in VR systems is ambiguous.

The objective of the sensory fidelity of VR technology is known as immersion. Presence refers to a user’s subjective perception of being present in the VR environment, even though they are physically located in another place. The power of the user to navigate virtual environments and their freedom to modify the environments is termed interactivity. Slater, Linakis, Usoh and Kooper [9] experimentally assessed and quantified the correlation between immersion, presence, and performance, demonstrating that a higher level of immersion and sense of presence can potentially increase people’s performance.

Based on the understanding of immersion, researchers generally recognise three types of VR systems: non-immersive systems (i.e., a virtual environment presented on a flat screen), semi-immersive systems (i.e., a room-based system), and fully immersive systems (i.e., a head-mounted display (HMD)) [11], with the intended purpose of triggering and supporting people’s imagination, attention, and instinctive behaviours. Additionally, the relationship between immersion, presence, and interactivity studied by Mütterlein [10] reveals that the level of immersion and presence can impact satisfaction with a VR experience, clearly indicating that immersion is an appropriate predictor in the VR context.

In numerous fields, the benefits of VR emerge from its power to build identifiable immersive experiences that motivate research and applications, including building planning, medical training, entertainment, design decision-making, data visualisation, and marketing [12,13,14,15,16,17,18]. Moreover, a series of recent clinical research that utilised immersive VR technology to deliver aversion therapy (i.e., specific phobias, social anxiety, post-traumatic stress disorder, depression, eating disorders, and paranoia assessment) has revealed that the results of VR treatments are equal to or more positive than those of traditional treatment methods [19,20,21,22,23,24,25,26,27,28,29,30]. In other words, repeated, controlled exposure to a computer-generated VR environment can provide individuals with a measured simulation of different problems that they face, which can help them to address and develop resilience to their aversions.

VR technology also presents another opportunity. Several works focussing on visual stimuli have investigated the restorative effects of VR environments used to induce positive emotions and behaviours based on attention restoration theory (ART) [31,32,33,34]. Researchers suggest that exposing participants to simulated natural environments has equal or greater effects compared to exposure to real-life environments. Exposure of participants who have undergone stressful events to these VR environments has positive effects, including stress relief, a reduction in cognitive fatigue, and a decrease in other negative effects [35,36]. Such evidence supports the usage of these restorative, simulated environments in future healthcare strategies to prompt more digital applications for well-being, entertainment, and virtual working environments. The foremost technical emphasis regarding immersive VR technology is likely to be on the visual fidelity of the stimuli, resulting in a higher level of immersion [1,2,3].

Colour, as the core element of visual experience, has been proven in prior studies to positively affect people’s cognitive functions [8,37], perceptions [38], psychological and emotional reactions, and, ultimately [4,39,40], behavioural intentions [41,42,43,44]. The human eye and brain work together to translate light into colour. The eye is the first part of the visual system. Specifically, the human eyeball contains three layers: the sclerotic, choroid, and retinal [45]. The retina itself consists of three layers: the retinal ganglion cells, bipolar cells, and two groups of receptors, known as rods and cones [46]. Colour vision starts with light passing via the cornea and the lens, which produce a clear image on the retina. The second element in vision is the brain, which controls the nervous system. The visual information (electrical signals) from the retina is sent via the optic nerve to the lateral geniculate nucleus of the thalamus and to the primary visual cortex, which ultimately processes the image and allows people to see both the real and virtual worlds. Numerous published findings have revealed a well-defined connection between human emotional reactions and certain colours, and this parallels the relationship between the influence of colours on people’s arousal and impulse levels [7]. More specifically, arousal refers to the physiological and psychological state of being awake. It is essential for the regulation of the psychological functions of attention, alertness, information processing (decision making or judgements), emotions, memory, and consciousness [47,48,49]. Impulsiveness is defined as a behavioural ability used to respond quickly and without mental reflection, which is associated with the control of a series of emotions and thoughts and cognitive performance [50].

Therefore, the design potential of colours must be considered particularly important in fundamental immersive technology design (i.e., virtual reality, augmented reality, and mixed reality). There is a considerable amount of documentation that relates to the immersive experience. For example, Brown and Cairns’ [51] research was concerned with a ground investigation of game immersion. They highlighted engagement, attention, and atmosphere as the keys to immersion. Their argument also highlights that the more attention and effort invested, the more immersed a gamer can feel. In line with colour and light research, an interesting question arises as to whether the effective use of colour and light can help create a higher degree of focus and concentration, potentially having a positive impact on immersion. There has been research concerning the use of colour to improve the immersive power of video games [52,53]. However, it is difficult to ascertain whether it is probable that the influence of colour and light on emotions and cognitive behaviour can positively impact immersion in VR. This paper focusses on this aspect of colour and cognition in VR. Douglas and Hargadon [54] proposed that the pleasure of immersion is derived from absorption within a familiar schema. However, we argue that the psychological design potential of colour and light in VR offers a more literal interpretation of immersion that can better trigger ‘psychological absorption’. A review study [55] illustrates the mechanism of how VR works, a small portion of ‘what there is to see’. Our perceptual system recognises a full model of the room that we are in, and this suggests that VR provides sufficient cues for the human perceptual system to imagine ‘this is a room’ and ‘being present’ in the room. In fact, it has been argued that in VR, our model of the scene around us tends to drive the movement of our eyes rather than eye movement guiding our perceptual model of the scene [56]. The design of VR environments, in some ways, is related to the design of viewing cabinets. Chernyak and Stark’s [56] research does not mention the potential of colour and light conditions as key visual cues to trigger people’s perceptual systems or their psychological and physiological reactions.

Considering the understood importance of colour and light as triggers of human response and engagement, an interesting question arises in this study as to the effective use of colour and light in VR and whether they can help create a higher degree of focus and concentration, potentially having a positive impact on immersion. Intriguingly, an immersive colour experience in VR could enhance people’s performance with cognitive tasks compared to the conventional colour imaging displays used in previous studies [4,37,38,39,57,58,59,60].

In the next section, we present comparative psychological experiments that validate the impact of colour on arousal and impulse levels in both physical and VR environments as an indirect approach to validating the impacts of colour on people’s cognitive abilities in reality and in VR spaces.

## 2. Experimental

### 2.1. Materials

**Figure 1:**
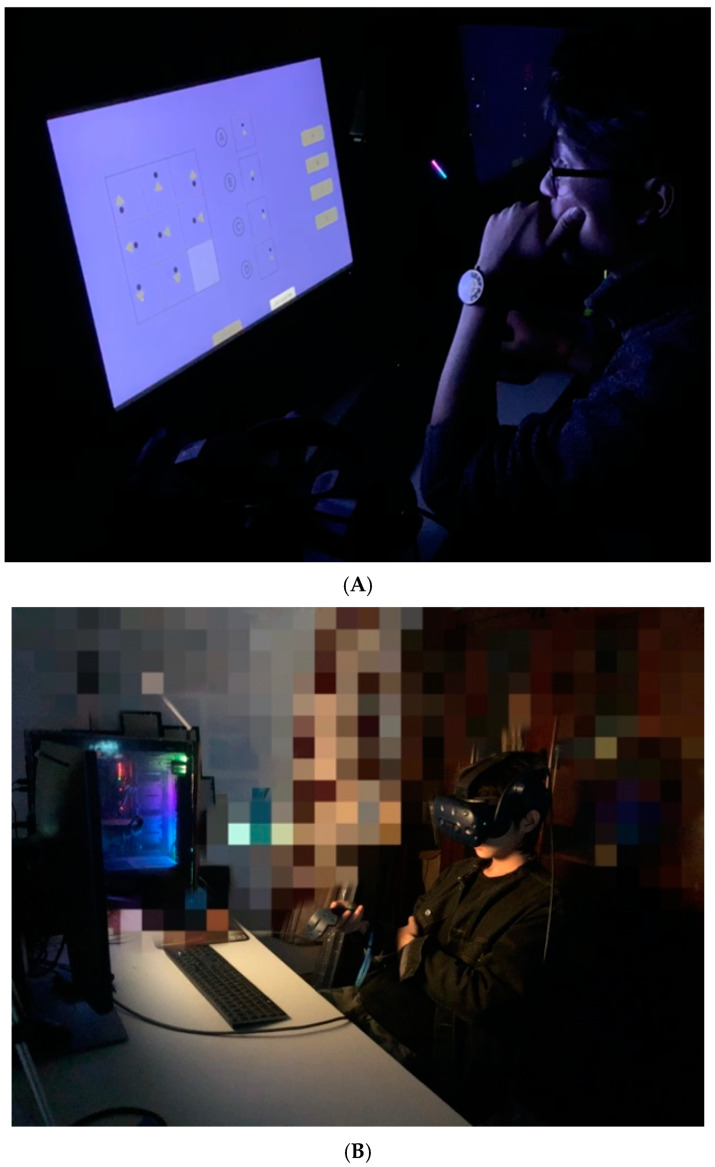
(**A**) Individual participant using the mouse to complete each question; (**B**) individual participant performing psychometric tasks using HTC Vive VR headset.

The experiments were performed using the Unity engine, HTC Vive Pro head-mounted display (HMD) (Bolton, UK), and a Dell desktop computer (Round Rock, TX, USA) (Figure 1A,B).

### 2.2. Colour Conditions

The experiments used seven background colours (i.e., reference white, red, purple, orange, yellow, green, and blue) from the Adobe hue, saturation, and brightness (HSB) colour system [7,61,62,63,64], with the equal luminosity setting adjusted. Specifically, the background colour of each question was defined by red, green, and blue (RGB) values, and the actual colours that were displayed on both the monitor and the VR headset were measured using an X-Rite i1Pro (Grand Rapids, MI, USA) (a professional-level spectral colour measurement instrument) in a dark laboratory environment. The colour measurement, the preliminary experiment, and the main experiment were all carried out 60 min after both the monitor and VR headset were turned on. The characteristics of the background colours in both the monitor and VR headset are reported in Appendix A. A preliminary experiment to validate the HSB consistency between the real-world and VR environments was carried out with 70 participants in the same dark laboratory environment before the main experiment. This involved 35 participants viewing the seven coloured backgrounds on the monitor first, followed by the VR headset, and the other 35 participants viewing the coloured backgrounds through the VR headset first and then on the monitor. After completing these observations, participants were asked to complete HSB consistency surveys, and the results (Appendix B) confirmed that the HSB values used in the main experiment were approximately constant between the monitor and VR headset. Only participants scoring above the average score were chosen for the main experiment.

### 2.3. Psychometric Tests

Psychometric testing methods were used to validate participants’ cognitive performance, as they are easily quantifiable and can feasibly be performed in both real-world and VR environments. The experiments employed six types of psychometric tests to measure participants’ logical thinking ability (logical rule and mathematics sequence tests), lateral thinking ability (spatial structure and rotation tests), and attention to detail (odd one out and same detail tests) [7,65,66,67,68] (see Table 1). The colours of the backgrounds (see Appendix C) and the order of presentation of the questions were randomised (for each participant). However, within each test, each participant was presented with a question with each of the seven coloured backgrounds. Note, however, that for different participants, the coloured backgrounds assigned to the questions within a test were different. The purpose of this was to ensure that if one of the questions was slightly more difficult than another, it would be equally likely to have any of the backgrounds for a participant and would remove bias. Response time and error rate were the two main parameters measured during the experiments. The results of these tests were used to estimate the levels of arousal and impulsiveness shown by each participant, which can be used as an indirect way to understand how colour affects cognitive ability [7,8,65].

### 2.4. Participants

A total of 70 participants (35 males and 35 females, aged between 20 and 28 years old) were recruited for the comparative experiments. To avoid variable cultural effects and the possibility that some participants might be more logical in their approach, all participants were Chinese undergraduate and postgraduate students randomly selected from the School of Media, Harbin Normal University.

### 2.5. Experimental Procedure

The comparative experiments were carried out in a dark room with separate participants. Participants were divided into two groups, with 35 assigned to the group in the physical environment (PE) using the conventional non-immersive colour imaging display and the other 35 assigned to the immersive virtual reality (VR) group. In both the PE and VR experimental sessions, all participants were required to complete the Ishihara colour vision test before entering the room to confirm that they had normal colour recognition ability [70]. After passing the test, the instructions for the entire experimental procedure were given to each participant, followed by a sample task including each type of psychometric test to familiarise participants with the process before beginning the main experiment. As a standard, participants were allowed to adjust to the reference white background picture for five minutes to compensate for chromatic adaption with participants in the VR group using the HMD and those in the PE group using a computer monitor. The main experiment started five minutes after they had adapted to the experimental conditions. Each participant was seated at a fixed distance of around 70 cm from a monitor with an aspect ratio of 16:9, and the design of the VR display gave an equivalent perspective to the PE environment.

## 3. Results

**Figure 2:**
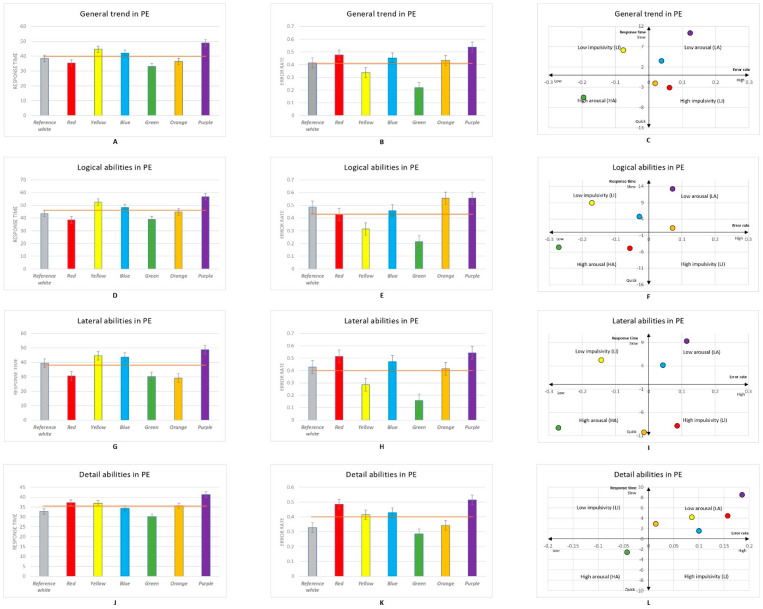
(**A**) General trend of response time by background colour in physical environment (PE); (**B**) general trend of error rate by background colour in PE; (**C**) colour impacts on general performance in PE visualised in the Error-Speed space; (**D**) response time of participants’ performance in logical abilities by background colour in PE; (**E**) error rate of participants’ performance in logical abilities by background colour in PE; (**F**) colour impacts on logical abilities in PE visualised in the Error-Speed space; (**G**) response time of participants’ performance in lateral abilities by background colour in PE; (**H**) error rate of participants’ performance in lateral thinking abilities by background colour in PE; (**I**) colour impacts on lateral thinking abilities in PE visualised in the Error-Speed space; (**J**) response time of participants’ performance in detail abilities by background colour in PE; (**K**) error rate of participants’ performance in detail abilities by background colour in PE; (**L**) colour impacts on detail abilities in PE visualised in the Error-Speed space. The bars represent mean changes, while the error bars are the standard error of the mean across individual participants. People’s responses to the reference white background are located on the centre axis in figures (**C**,**F**,**I**,**L**).

**Figure 3:**
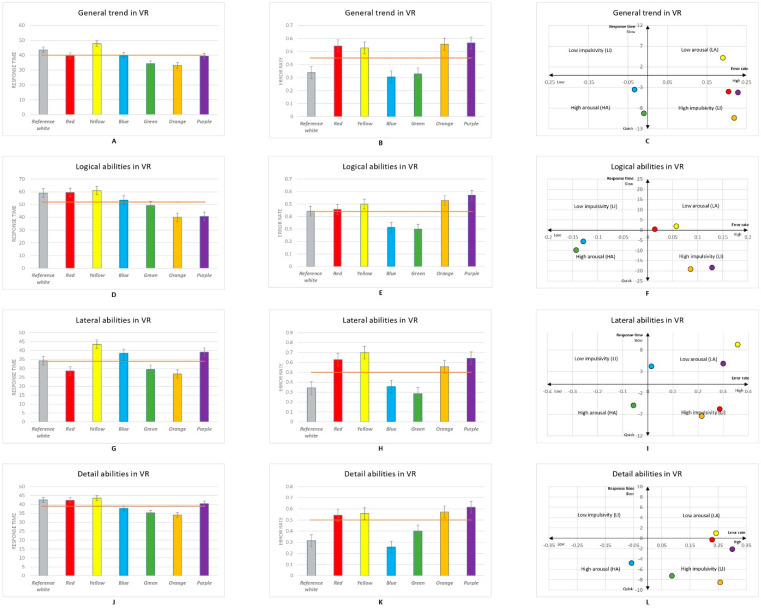
(**A**) General trend of response time by background colour in virtual reality (VR); (**B**) general trend of error rate by background colour in VR; (**C**) colour impacts on general performance in VR visualised in the Error–Speed space; (**D**) response time of participants’ performance in logical abilities by background colour in VR calculated in seconds; (**E**) error rate of participants’ performance in logical abilities by background colour in VR; (**F**) colour impacts on logical abilities in VR visualised in the Error–Speed space; (**G**) response time of participants’ performance in lateral thinking abilities by background colour in VR; (**H**) error rate of participants’ performance in lateral abilities by background colour in VR; (**I**) colour impacts on lateral thinking abilities in VR visualised in the Error–Speed space; (**J**) response time of participants’ performance in detail abilities by background colour in VR; (**K**) error rate of participants’ performance in detail abilities by background colour in VR; (**L**) colour impacts on detail abilities in VR visualised in the Error–Speed space. The bars represent mean changes, while the error bars are the standard error of the mean across individual participants. People’s responses to the reference white background are located on the centre axis in figures (**C**,**F**,**I**,**L**).

**Figure 4:**
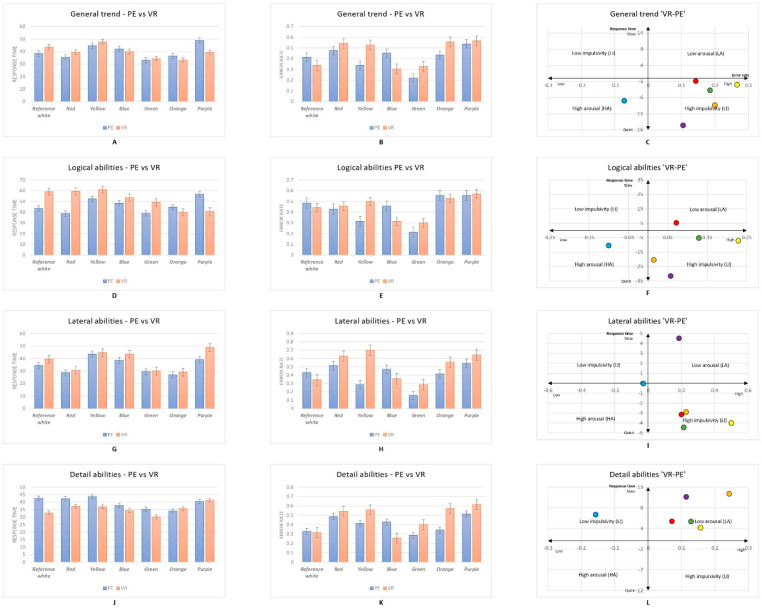
(**A**) General trend of response time by background colour in PE and VR; (**B**) general trend of error rate by background colour in PE and VR; (**C**) colour impacts (in VR compared with PE) on general performance visualised in the Error–Speed space; (**D**) response time of participants’ performance in logical abilities by background colour in PE and VR; (**E**) error rate of participants’ performance in logical abilities by background colour in PE and VR; (**F**) colour impacts (in VR compared with PE) on logical abilities in VR visualised in the Error–Speed space; (**G**) response time of participants’ performance in lateral thinking abilities by background colour in PE and VR; (**H**) error rate of participants’ performance in lateral thinking abilities by background colour in PE and VR; (**I**) colour impacts (in VR compared with PE) on lateral thinking abilities in VR visualised in the Error–Speed space; (**J**) response time of participants’ performance in detail abilities by background colour in PE and VR; (**K**) error rate of participants’ performance in detail abilities by background colour in PE and VR; (**L**) colour impacts (in VR compared with PE) on detail abilities in VR visualised in the Error–Speed space. The bars represent mean changes, while the error bars are the standard error of the mean across individual participants. People’s responses to the reference white background are located on the centre axis in figures (**C**,**F**,**I**,**L**). VR: virtual reality; PE: Physical environment.

Seventy participants took part in the experiment. A total number of 2948 responses were recorded, with 420 responses per colour obtained from participants across both the PE and VR experimental sessions. Statistical analysis was performed using Statistical Product and Service Solutions (IBM Corp. Released 2020. IBM SPSS Statistics for Windows, Version 27.0, Armonk, NY, USA) software. Figure 2, Figure 3 and Figure 4 show the mean averages of the response time and error rate pooled from all six types of psychometric tests in both the PE and VR environments. The Kruskal–Wallis test and an independent *t*-test were used to analyse the data obtained from the experiment. Participants’ impulsiveness and arousal were defined as: high arousal (HA), faster reactions and lower error rate; low arousal (LA), slower reactions and higher error rate; high impulsiveness (HI), shorter response time and higher error rate; and low impulsiveness (LI), longer response time and lower error rate (all compared with the mean of the reference white colour background) [7,8,65,71].

### 3.1. Colour Stimuli in the Reality Session

According to the data obtained from the psychometric tests, the green background generally resulted in both the fastest response time and the lowest error rate, while the purple background generally resulted in both the slowest response time and the highest error rate (Figure 2A,B). These findings suggest that participants experienced the highest state of arousal when viewing the green background and the lowest state of arousal when viewing the purple background. The Kruskal–Wallis test revealed significant differences in the error rate between the reference white and green (*p* = 0.001), red and green (*p* < 0.001), yellow and purple (*p* < 0.001), blue and green (*p* < 0.001), green and orange (*p* < 0.001), and green and purple (*p* < 0.01) backgrounds. There were also significant differences in response time between orange and green (*p* = 0.04), between blue and purple (*p* = 0.019), and between the purple and yellow (*p* = 0.004), reference white (*p* = 0.051), and blue (*p* = 0.004) backgrounds (see Appendix D). When considering both the error rate and response time (Figure 2C), the results suggest that participants’ overall cognitive abilities can be influenced by colours. Specifically, participants experienced an HA state when they completed questions with the green background, and they experienced an LA state with the blue and purple backgrounds. Additionally, for the red and yellow backgrounds, participants were shown to experience an HI state when they were completing the questions. When viewing the yellow background, participants made fewer errors and responded more slowly, suggesting that it caused participants to experience an LI state.

Participants’ logical ability was measured using the logical rule and mathematics sequence tests. Unlike the general trend, the results shown in Figure 2D suggest that participants reacted fastest when viewing the red background and slowest when viewing the purple background; however, the Kruskal–Wallis test showed no significant difference in participants’ response times during logical ability tasks. As for the error rate (Figure 2E), participants viewing the green background made the fewest errors, while participants viewing the orange and purple backgrounds gave the most incorrect answers to logical ability questions. Specifically, significant differences were found between the reference white and green (*p* = 0.017), yellow and orange (*p* = 0.047), and yellow and purple (*p* = 0.001) and between the purple and green (*p* = 0.004) and red backgrounds (*p* = 0.007). Together with the results of response time and error rate, these findings suggest that participants’ logical ability can be significantly impacted by colours (Figure 2F). Specifically, the green and red backgrounds can cause HA effects on participants’ logical abilities, and the purple and orange backgrounds can cause LA effects on logical abilities. For the yellow and blue backgrounds, participants experienced an LI state when completing tests that required logical abilities.

The results of the spatial structure and rotation tests suggest that colour can affect participants’ lateral thinking abilities. As shown in Figure 2G, participants viewing the orange background gave the fastest responses, and those viewing the purple background gave the slowest responses; however, the impacts of colour on participants’ response times were not found to be significant. Participants made the fewest errors when viewing the green background and the most errors when viewing the purple background (Figure 2H). The Kruskal–Wallis test revealed significant differences between the reference white and green (*p* = 0.014), red and green (*p* < 0.01), yellow and purple (*p* = 0.025), green and blue (*p* = 0.002), green and orange (*p* = 0.025), green and purple (*p* < 0.05), and orange and blue (*p* = 0.025) backgrounds (see Appendix E). Figure 2I shows the distribution of the six colours in relation to response time and error rate. These data indicate that participants’ lateral thinking ability was affected by the green and orange backgrounds, which caused an HA state, and the blue and purple backgrounds, which caused an LA state. Moreover, participants’ lateral thinking ability was influenced by the red background, causing an HI state, and the yellow background, inducing an LI state.

The impacts of colour on participants’ attention to detail were measured by odd one out and same detail tests. Participants viewing the purple background had the slowest response times and made the most errors, whereas those viewing the green background had both the fastest response time and lowest error rate (Figure 2J,K); however, these differences in attention to detail were not found to be significant. The effects of the colours on participants’ attention to detail are shown in Figure 2L, with orange, yellow, blue, and red causing an LA state and green causing an HA state.

### 3.2. Colour Stimuli in VR

From Figure 3A,B, it can be observed that there was a general impact of colour viewed in VR on the response time and error rate during the tasks. Participants viewing the green background gave the fastest responses, while participants viewing the yellow background gave the slowest response. The Kruskal–Wallis test revealed significant differences in participant response time between the yellow and green (*p* < 0.01), orange (*p* < 0.01), and reference white (*p* = 0.002) backgrounds and between yellow and red (*p* = 0.002) and blue (*p* = 0.014), as well as red and reference white (*p* = 0.03), backgrounds. Participants made the fewest errors when viewing the blue background, while participants viewing the purple background made the most errors. The Kruskal–Wallis test showed significant differences between the reference white and red (*p* < 0.001), reference white and yellow (*p* = 0.001), reference white and orange (*p* < 0.001), red and blue (*p* < 0.01), red and green (*p* < 0.01), yellow and blue (*p* < 0.001), yellow and green (*p* = 0.001), blue and orange (*p* < 0.01), blue and purple (*p* < 0.001), green and orange (*p* < 0.001), and green and purple (*p* < 0.001) backgrounds (see Appendix F). Both the response time and error rate (Figure 3C) suggest that participants viewing the green and blue backgrounds experienced an HA state, and the yellow background caused an LA state. In addition, participants viewing the red, purple, and orange backgrounds seemed to react more quickly and make more errors, suggesting that high impulsivity affected participants’ general performance in VR.

Figure 3D,E display the impact of colour in VR on logical ability. Participants reacted fastest when viewing the orange background and slowest when viewing the yellow background. Significant differences in response time were found between green and yellow (*p* = 0.017), orange (*p* = 0.007), and purple (*p* = 0.001) backgrounds, as well as between blue and yellow (*p* = 0.027), orange (*p* = 0.011), and purple (*p* = 0.002) backgrounds. Figure 3E shows that viewing the green background led to the lowest error rate, while viewing the purple background led to the highest error rate. The Kruskal–Wallis test indicated significant differences between the blue and purple (*p* = 0.002), orange (*p* = 0.011), and yellow (*p* = 0.027) backgrounds, as well as green and purple (*p* = 0.001), yellow (*p* = 0.017), and orange (*p* = 0.007) backgrounds. When considering both the response time and error rate, Figure 3F shows that blue and green backgrounds can lead to an HA state when participants are tested for their logical ability, while the red and yellow backgrounds cause an LA state. Orange and purple in VR can lead to an HI effect on logical ability.

The results of the spatial structure and rotation tests suggest that colour stimuli delivered via VR technology can significantly influence participants’ lateral thinking ability. Figure 3G shows that participants responded fastest when viewing the orange background and slowest when viewing the yellow background. The Kruskal–Wallis test revealed a significant difference in response times between red and blue (*p* = 0.046), purple (*p* = 0.031), and yellow (*p* = 0.002) backgrounds, as well as between the orange and yellow (*p* = 0.031) and purple (*p* = 0.048) backgrounds, and between green and yellow (*p* = 0.005) backgrounds. As for the error rate, Figure 3H shows that when viewing the green background, participants had the lowest error rate, and those viewing the yellow background had the highest error rate. Significant differences in error rates during lateral thinking ability tests were found between the reference white and yellow (*p* = 0.009), red (*p* = 0.001), and purple (*p* < 0.001) backgrounds, red and green (*p* = 0.001), yellow and blue (*p* = 0.015), yellow and green (*p* = 0.001), blue and purple (*p* = 0.001), and orange and blue (*p* = 0.018) backgrounds (see Appendix F). In summary, Figure 3I shows that when performing lateral thinking ability tests, green led to the highest state of arousal; yellow, blue, and purple led to the greatest decrease in arousal; and orange and yellow led to an increase in impulsivity.

Further analysis of the influence of colour stimuli on participants’ attention to detail shows that participants reacted slowest when viewing the yellow background and fastest when viewing the orange background (Figure 3J); significant differences were found between the green and yellow (*p* = 0.013), purple (*p* = 0.009), and reference white (*p* = 0.004) backgrounds. In regard to the error rate, Figure 3K shows that participants viewing the blue background made the fewest errors, while participants viewing the purple background had the highest error rate. Specifically, the Kruskal–Wallis test indicated that significant differences were found between the reference white and red (*p* = 0.007), orange (*p* = 0.002), purple (*p* < 0.001), and yellow (*p* = 0.004) backgrounds, between red and blue (*p* = 0.010), yellow and blue (*p* < 0.001), blue and orange (*p* < 0.001), and blue and purple (*p* < 0.001), and between green and purple (*p* = 0.011) backgrounds, suggesting that all six colours significantly affected participants’ attention to detail. In summary, Figure 3L shows that blue increased the aroused state, yellow decreased the aroused state, green and orange gave rise to increased impulsivity, and although red is located on the boundary of the HI quadrant, it also appears to increase impulsivity.

### 3.3. Comparative Analysis of Colour Stimuli between the PE and VR

A comparative analysis of the impact of colour stimuli between the PE and VR environments is displayed in Figure 4A–C. An independent *t*-test was used to calculate the significant differences in the data. Overall, the effects of colour delivered via VR technology appear to have a greater role in increasing impulsivity compared to the PE, and blue also appears to have a greater impact on increasing arousal in participants using VR compared to the PE. Specifically, independent *t*-tests revealed significant differences in error rates between the reality and VR experimental sessions in yellow (*p* < 0.001), blue (*p* = 0.002), green (*p* = 0.012), and orange (*p* = 0.011). This suggests that in the VR environment, participants experienced significantly increased arousal when viewing the blue background and significantly increased impulsivity when viewing the purple, green, orange, red, and yellow backgrounds.

The results in Figure 4D–F show the impact of colour on participants’ logical abilities in both the PE and VR (see Appendix G). In VR, participants experienced increased impulsivity when viewing the purple, orange, green, and yellow backgrounds, an increased state of arousal when viewing the blue background, and a decreased state of arousal when viewing the red background. In addition, there were significant differences between the PE and VR in participants’ response times when viewing the red (*p* = 0.023), orange (*p* = 0.047), and purple (*p* = 0.040) backgrounds and in error rates when viewing the yellow (*p* = 0.001), blue (*p* = 0.003), and green (*p* = 0.022) backgrounds.

With regard to the lateral thinking ability between the PE and VR, generally, participants had higher impulsive states when viewing green, red, orange, and yellow backgrounds in VR. Interestingly, the purple background in VR seems to have significantly lowered the state of arousal during lateral thinking ability tests compared to that in the PE. Moreover, blue is located on the boundary of the four quadrants, with seemingly no difference in lateral thinking ability when the blue background was viewed either in the PE or VR. Significant differences were found between the PE and VR in the response times when participants viewed the orange background (*p* = 0.049) and error rates when viewing the red (*p* = 0.032), blue (*p* = 0.021), green (*p* < 0.001), and purple (*p* = 0.033) backgrounds.

Figure 4J–L show the impact of colour on attention to detail between the PE and VR. Interestingly, in VR, the effects of colour appear to have a lower effect on arousal compared with the PE, except blue seems to have a lower impact on impulsivity. Specifically, significant differences were found between the error rates in the PE and VR when viewing the blue (*p* < 0.001) and green (*p* = 0.007) backgrounds (see Appendix H).

## 4. Discussion

This study is concerned with exploring the design potential of colours to influence people’s cognitive abilities (i.e., logical, lateral, and detail abilities) in VR. In this study, we compared people’s cognitive responses to various colours between reality and VR laboratory settings. The data collected from the comparative psychological experiments partly support our two hypotheses.

First, people’s performances on cognitive tasks were significantly affected by colour, which is in line with previous findings [4,37,38,39,57,58,59,60]. In terms of the general impact in a reality setting, green seems to cause the most arousal in people, and purple results in the lowest arousal. This is partly in agreement with a previous study by Ciccone [72] that indicates that short-wavelength lights (i.e., blue and green) seem to have a higher arousal effect. However, some studies indicate that green seems to cause a relaxing and calming effect (relatively low arousal effect) [7,73]. One possible explanation for this could be that the chroma and brightness conditions used in the experiment remained at an optimal level. According to the Yerkes–Dodson law, increased levels of arousal can increase performance to a positive level, while, if below the optimum, an increased level of arousal is followed by negative performance [74]. In line with our results, the green colour used in this study is likely to raise arousal to an optimal level compared with the other six hues. Moreover, our results show that yellow is located in the LI quadrant, while orange and red are in the HI quadrant. These findings are somewhat in agreement with Duan, Rhodes, and Cheung [7], whose work indicates that blue and yellow induce people to produce more errors. However, we discovered that green is located in the HA quadrant and purple is in the LI quadrant, differing from Duan, Rhodes, and Cheung [7]. The results from the psychological experiments show evidence that colour can have different impacts on logical, lateral, and detail abilities. As to logical abilities, blue is located in the LI quadrant, red is in the HA quadrant, and orange is in the LA quadrant. This suggests that blue seems to cause lower impulsive effects, red causes higher arousal effects, and orange causes lower arousal effects on tests that require logical abilities. When it comes to the test that requires lateral thinking abilities, blue is in the LA quadrant, red is in the HI quadrant, and orange is in the HA quadrant, suggesting that the colour impact on lateral and logical thinking abilities is somewhat different. Interestingly, since the detail ability tests required participants to think logically (holistic perception) and laterally (detail-oriented), in our research, almost all colours (orange, yellow, blue, red, and purple) are located in the LA quadrant, and only green is in the HA quadrant. This shows that green may have a higher arousal effect on detail abilities, and orange, yellow, blue, red, and purple may have less influence on arousal in detail abilities.

Second, our research shows that the psychological impact of colour stimulation on people’s cognitive performance can be obtained in a VR environment, which would demonstrate the design potential of colour for building positive immersion in VR. However, the impact of colour stimulation on cognitive abilities in VR is inconsistent with what we found in the reality session. Generally, green and blue are located in the HA quadrant, yellow is in the LA quadrant, and orange, red, and purple are in the LI quadrant. Compared with the general colour effects in the reality session, only green, orange, and red are relatively consistent with those found in the reality session. Similar to the reality session, in VR, the colour influences on logical and lateral thinking vary. Concerning colour influence on detail abilities in VR, nearly all colours seem to have a higher impulsive influence, except for blue in the HA quadrant and yellow in the LA quadrant. Collectively, in both the reality and VR sessions, the most stable colour was green, which caused higher arousal effects on participants’ cognitive abilities. Comparative analyses of the colour stimuli between reality and VR generally reveal that colour stimulations in VR seem to have higher impulsive effects than in reality. Only with tests that required detail abilities did the colour stimuli in VR seem to have less influence on arousal than in the reality session.

The contributions of this study demonstrate that colour is critical in influencing people’s cognitive abilities in both reality and VR environments. These findings could be used in various design areas, including in product design by using colours to engage buying behaviours and in environmental design by using colours to motivate people’s performance. In an era of immersive technology design, our work suggests that the effective use of colour may somehow create positive effects on immersion. This indicates that VR, as a new design technology, will be a positive trigger of designers’ creativity and coherence. Simultaneously, some limitations have to be considered, especially the restrictions of using an HMD headset (i.e., discomfort). In addition, a replica of the VR environment strengthens the colour stimuli to help create a higher degree of focus and concentration, which therefore offers some potential to make colour stimulation stronger than in the reality session. The brightness conditions of this experiment were adjusted based on the VR headset. The participant selection criteria (exclusively Chinese students), although designed to ensure consistency, could be considered to be a study limitation. Finally, the different interactive functions between a mouse and joysticks may have an impact on people’s response time, although both are standard touch-button interfaces.

## Figures and Tables

**Table 1:** Functions of six types of psychometric tests used in the experiments.

Cognitive Abilities	Tests
Logical thinking abilities	Logical ability tests [7,65,69]: Logical rule test Mathematics sequence
Lateral thinking abilities (also known as spatial imagination abilities or lateral abilities in the study)	Spatial imagination ability tests [7,65,69]: Spatial structure test Rotation test
Both logical abilities and lateral thinking abilities (also known as detail abilities in this study)	Detail ability tests [7,66]: Odd one out Same detail test

**Figure A1:**
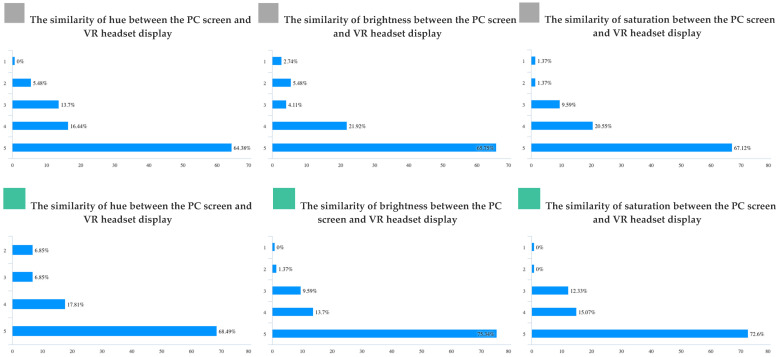

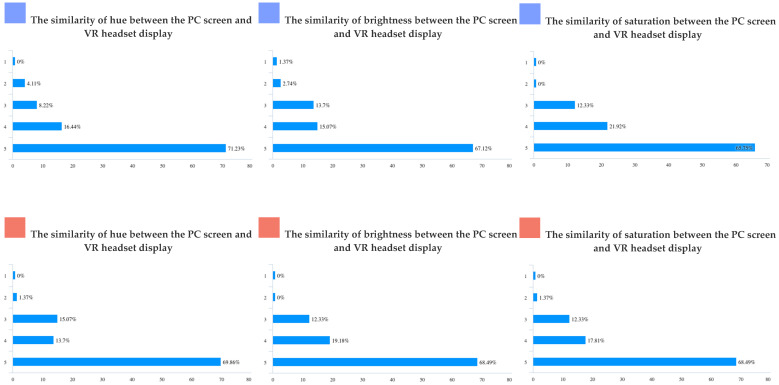

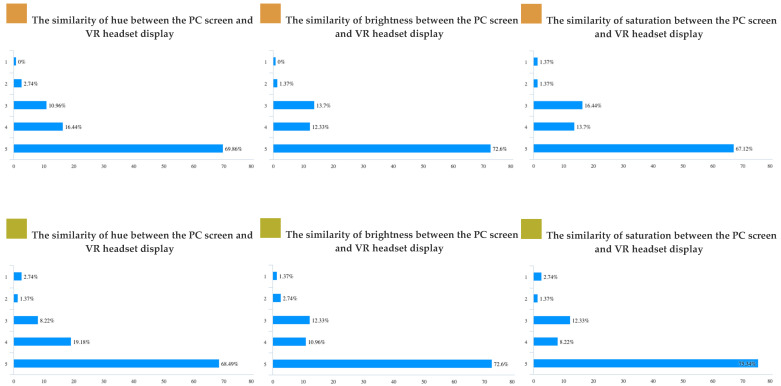

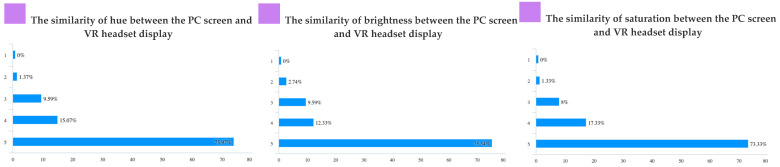
People’s perception of the similarity of hue, brightness and saturation between the reality and virtual reality (1 = Dissimilar, 2 = Slightly dissimilar, 3 = Slightly similar, 4 = Similar, 5 = Same).

**Figure A2:**
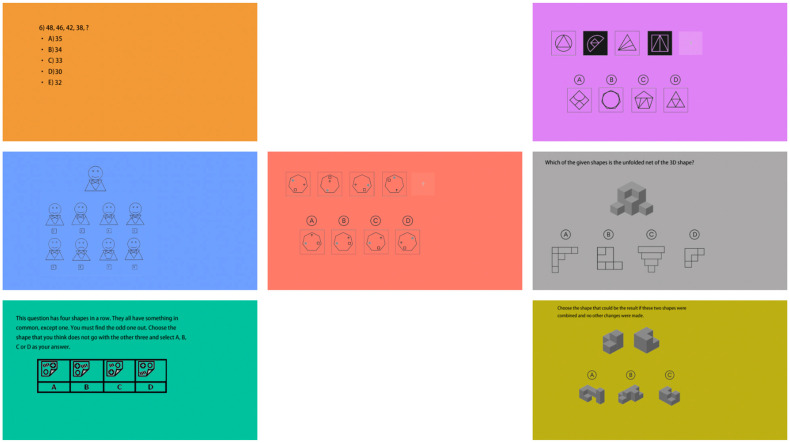
Examples of questions and 7 coloured backgrounds.

**Figure A3:**
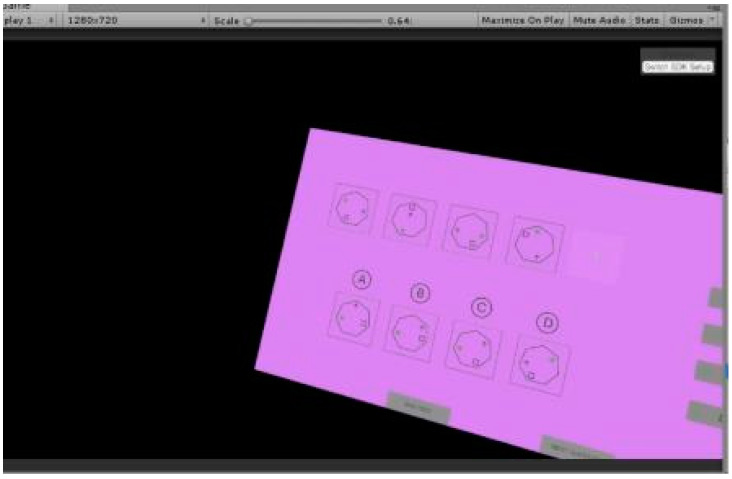
Examples of experimental setup.

## Data Availability

The data presented in this study are available on request from the corresponding author.

## Appendix A

**Table A1:** The characteristics of the background colours of the monitor.

Colours	L*	C*	h	a*	b*	R	G	B
Visual Reference White	70.01	0.51	28.34	0.24	0.19	171.27	170.01	169.86
Red	69.42	69.08	34.33	23.95	35.01	244.31	121.54	103.56
Yellow	70.52	69.13	99.63	−25.59	55.21	187.82	175.99	19.87
Blue	69.77	65.86	286.26	33.33	−36.64	110.09	158.86	255.00
Green	67.89	67.26	177.63	−54.76	−3.48	62.76	193.52	156.59
Orange	68.29	67.66	67.84	−0.57	56.18	242.31	154.31	55.88
Purple	68.01	68.17	320.95	48.07	−22.74	221.76	129.76	243.86

Note: L*: perceptual lightness; C*: chroma; h: hue; a* and b*: four unique colors of human vision: red, green, blue, and yellow; R: red; G: green; B: blue.

**Table A2:** The characteristics of the background colours within the VR headset.

Colours	L*	C*	h	a*	b*	R	G	B
Visual Reference White	72.12	0.49	29.67	0.22	0.19	171.27	170.01	169.86
Red	70.31	70.34	32.83	25.31	34.33	244.31	121.54	103.56
Yellow	70.36	68.05	97.79	−23.83	54.80	187.82	175.99	19.87
Blue	69.32	64.87	288.17	34.01	−35.66	110.09	158.86	255.00
Green	68.89	67.15	178.62	−54.11	−4.18	62.76	193.52	156.59
Orange	69.46	67.66	69.21	−1.63	55.99	242.31	154.31	55.88
Purple	70.57	67.63	321.05	47.39	−22.38	221.76	129.76	243.86

Note: L*: perceptual lightness; C*: chroma; h: hue; a* and b*: four unique colors of human vision: red, green, blue, and yellow; R: red; G: green; B: blue.

## Appendix C

Examples of questions and 7 coloured backgrounds shown during the experiment in both the reality and virtual reality sessions.

## Appendix D

The Kruskal–Wallis test analysis of people’s responses to colours in PE (general effects).

**Table A3:** Effects on error rate (general effects).

Pairwise Comparisons of Colours (Error Rate)			
Sample 1–Sample 2	Test Statistic	Sig.	Adj. Sig. a
Reference White–Red	1.629	0.202	1
Reference White–Yellow	2.597	0.107	1
Reference White–Blue	0.621	0.431	1
Reference White–Green	18.496	0	0
Reference White–Orange	0.156	0.693	1
Reference White–Purple	6.453	0.011	0.233
Red–Yellow	8.296	0.004	0.083
Red–Blue	0.239	0.625	1
Red–Green	30.615	0	0
Red–Orange	0.778	0.378	1
Red–Purple	.b	.	.
Yellow–Blue	5.738	0.017	0.349
Yellow–Green	7.405	0.007	0.137
Yellow–Orange	4.02	0.045	0.945
Yellow–Purple	17.062	0	0.001
Blue–Green	25.634	0	0
Blue–Orange	0.154	0.694	1
Blue–Purple	3.086	0.079	1
Green–Orange	21.936	0	0
Green–Purple	45.432	0	0
Orange–Purple	4.613	0.032	0.666

Note: Each row tests the null hypothesis that the Sample 1 and Sample 2 distributions are the same. Asymptotic significances (2-sided tests) are displayed. The significance level is 0.050; a: Significance values have been adjusted by the Bonferroni correction for multiple tests; b: Unable to compute because all sample medians in this pair are less than or equal to the hypothesised median. Sig: means significance.

**Table A4:** Effects on response time (general effects).

Pairwise Comparisons of Colours (Response Time)			
Sample 1–Sample 2	Test Statistic	Sig.	Adj. Sig. a
Orange–Yellow	1.371	0.242	1
Orange–Red	4.61	0.032	0.668
Orange–Green	4.2	0.04	0.849
Orange–Blue	5.486	0.019	0.403
Orange–Reference White	2.438	0.118	1
Orange–Purple	11.667	0.001	0.013
Yellow–Red	0.152	0.696	1
Yellow–Green	0.61	0.435	1
Yellow–Blue	0.467	0.495	1
Yellow–Reference White	0.467	0.495	1
Yellow–Purple	4.2	0.04	0.849
Red–Green	0.238	0.626	1
Red–Blue	0.343	0.558	1
Red–Reference White	0.343	0.558	1
Red–Purple	3.086	0.079	1
Green–Blue	0.152	0.696	1
Green–Reference White	0.343	0.558	1
Green–Purple	3.086	0.079	1
Blue–Reference White	0.152	0.696	1
Blue–Purple	4.2	0.04	0.849
Reference White–Purple	3.81	0.051	1

Note: Each row tests the null hypothesis that the Sample 1 and Sample 2 distributions are the same. Asymptotic significances (2-sided tests) are displayed. The significance level is 0.050. a: Significance values have been adjusted by the Bonferroni correction for multiple tests.

## Appendix E

The Kruskal–Wallis test analysis of people’s responses to colours in reality—logical, lateral and detail abilities.

**Table A5:** Effects on response time (logical thinking abilities).

Independent-Samples Kruskal–Wallis Test with Bonferroni Correction: Summary	
Total N	490
Test Statistic	2.926 a,b
Degrees Of Freedom	6
Asymptotic Sig. (2-sided test)	0.818

Note: a: The test statistic is adjusted for ties. b: Multiple comparisons were not performed because the overall test did not show significant differences across samples.

**Table A6:** Effects on error rate (logical thinking abilities).

Pairwise Comparisons of Colours_PE					
Sample 1–Sample 2	Test Statistic	Std. Error	Std. Test Statistic	Sig.	Adj. Sig. a
Orange–Green	10.286	23.934	0.43	0.667	1
Orange–Red	13.729	23.934	0.574	0.566	1
Orange–Blue	34.221	23.934	1.43	0.153	1
Orange–Reference_White	36.829	23.934	1.539	0.124	1
Orange–Yellow	47.55	23.934	1.987	0.047	0.986
Orange–Purple	−78.386	23.934	−3.275	0.001	0.022
Green–Red	3.443	23.934	0.144	0.886	1
Green–Blue	23.936	23.934	1	0.317	1
Green–Reference_White	26.543	23.934	1.109	0.267	1
Green–Yellow	37.264	23.934	1.557	0.119	1
Green–Purple	−68.1	23.934	−2.845	0.004	0.093
Red–Blue	−20.493	23.934	−0.856	0.392	1
Red–Reference_White	23.1	23.934	0.965	0.334	1
Red–Yellow	−33.821	23.934	−1.413	0.158	1
Red–Purple	−64.657	23.934	−2.701	0.007	0.145
Blue–Reference_White	2.607	23.934	0.109	0.913	1
Blue–Yellow	13.329	23.934	0.557	0.578	1
Blue–Purple	−44.164	23.934	−1.845	0.065	1
Reference_White–Yellow	−10.721	23.934	−0.448	0.654	1
Reference_White–Purple	−41.557	23.934	−1.736	0.083	1
Yellow–Purple	−30.836	23.934	−1.288	0.198	1

Note: Each row tests the null hypothesis that the Sample 1 and Sample 2 distributions are the same. Asymptotic significances (2-sided tests) are displayed. The significance level is 0.050. a: Significance values have been adjusted by the Bonferroni correction for multiple tests.

**Table A7:** Effects on response time (lateral thinking abilities).

Total N	490
Test Statistic	3.801 a,b
Degrees Of Freedom	6
Asymptotic Sig. (2-sided test)	0.704

Note: a: The test statistic is adjusted for ties. b: Multiple comparisons were not performed because the overall test did not show significant differences across samples. N: sample size; sig: significance.

**Table A8:** Effects on error rate (lateral thinking abilities).

Total N	490
Test Statistic	2.926 a,b
Degrees Of Freedom	6
Asymptotic Sig. (2-sided test)	0.818

Note: a: The test statistic is adjusted for ties. b: Multiple comparisons were not performed because the overall test did not show significant differences across samples.

**Table A9:** Effects on response time (attention to details).

Pairwise Comparisons of Colours_PE					
Sample 1–Sample 2	Test Statistic	Std. Error	Std. Test Statistic	Sig.	Adj. Sig. a
Orange–Green	10.286	23.934	0.43	0.667	1
Orange–Red	13.729	23.934	0.574	0.566	1
Orange–Blue	34.221	23.934	1.43	0.153	1
Orange–Reference_White	36.829	23.934	1.539	0.124	1
Orange–Yellow	47.55	23.934	1.987	0.047	0.986
Orange–Purple	−78.386	23.934	−3.275	0.001	0.022
Green–Red	3.443	23.934	0.144	0.886	1
Green–Blue	23.936	23.934	1	0.317	1
Green–Reference_White	26.543	23.934	1.109	0.267	1
Green–Yellow	37.264	23.934	1.557	0.119	1
Green–Purple	−68.1	23.934	−2.845	0.004	0.093
Red–Blue	−20.493	23.934	−0.856	0.392	1
Red–Reference_White	23.1	23.934	0.965	0.334	1
Red–Yellow	−33.821	23.934	−1.413	0.158	1
Red–Purple	−64.657	23.934	−2.701	0.007	0.145
Blue–Reference_White	2.607	23.934	0.109	0.913	1
Blue–Yellow	13.329	23.934	0.557	0.578	1
Blue–Purple	−44.164	23.934	−1.845	0.065	1
Reference_White–Yellow	−10.721	23.934	−0.448	0.654	1
Reference_White–Purple	−41.557	23.934	−1.736	0.083	1
Yellow–Purple	−30.836	23.934	−1.288	0.198	1

Note: Each row tests the null hypothesis that the Sample 1 and Sample 2 distributions are the same. Asymptotic significances (2-sided tests) are displayed. The significance level is 0.050. a: Significance values have been adjusted by the Bonferroni correction for multiple tests.

**Table A10:** Effects on error rate (attention to details).

Independent-Samples Kruskal–Wallis Test Summary	
Total N	490
Test Statistic	2.926 a,b
Degrees Of Freedom	6
Asymptotic Sig. (2-sided test)	0.818

Note: a: The test statistic is adjusted for ties. b: Multiple comparisons were not performed because the overall test did not show significant differences across samples.

## Appendix F

The Kruskal–Wallis Test analysis of people’s responses to colours in VR—General effects.

**Table A11:** Effects on error rate (general effects).

Pairwise Comparisons of Colours_Error Rate					
Sample 1–Sample 2	Test Statistic	Std. Error	Std. Test Statistic	Sig.	Adj. Sig. a
Blue–Green	−17.5	35.713	−0.49	0.624	1
Blue–Reference White	24.5	35.713	0.686	0.493	1
Blue–Yellow	164.5	35.713	4.606	0	0
Blue–Red	175	35.713	4.9	0	0
Blue–Orange	−185.5	35.713	−5.194	0	0
Blue–Purple	−192.5	35.713	−5.39	0	0
Green–Reference White	7	35.713	0.196	0.845	1
Green–Yellow	147	35.713	4.116	0	0.001
Green–Red	157.5	35.713	4.41	0	0
Green–Orange	−168	35.713	−4.704	0	0
Green–Purple	−175	35.713	−4.9	0	0
Reference White–Yellow	−140	35.713	−3.92	0	0.002
Reference White–Red	−150.5	35.713	−4.214	0	0.001
Reference White–Orange	−161	35.713	−4.508	0	0
Reference White–Purple	−168	35.713	−4.704	0	0
Yellow–Red	10.5	35.713	0.294	0.769	1
Yellow–Orange	−21	35.713	−0.588	0.557	1
Yellow–Purple	−28	35.713	−0.784	0.433	1
Red–Orange	−10.5	35.713	−0.294	0.769	1
Red–Purple	−17.5	35.713	−0.49	0.624	1
Orange–Purple	−7	35.713	−0.196	0.845	1

Note: Each row tests the null hypothesis that the Sample 1 and Sample 2 distributions are the same. Asymptotic significances (2-sided tests) are displayed. The significance level is 0.050. a: Significance values have been adjusted by the Bonferroni correction for multiple tests.

**Table A12:** Effects on response time (general effects).

Pairwise Comparisons of Colours_Response Time					
Sample 1–Sample 2	Test Statistic	Std. Error	Std. Test Statistic	Sig.	Adj. Sig. a
Green–Orange	−16.757	41.427	−0.405	0.686	1
Green–Red	35.89	41.427	0.866	0.386	1
Green–Blue	62.983	41.427	1.52	0.128	1
Green–Purple	−110.395	41.427	−2.665	0.008	0.162
Green–Reference white	125.862	41.427	3.038	0.002	0.05
Green–Yellow	164.445	41.427	3.97	0	0.002
Orange–Red	19.133	41.427	0.462	0.644	1
Orange–Blue	46.226	41.427	1.116	0.264	1
Orange–Purple	−93.638	41.427	−2.26	0.024	0.5
Orange–Reference White	109.105	41.427	2.634	0.008	0.177
Orange–Yellow	147.688	41.427	3.565	0	0.008
Red–Blue	−27.093	41.427	−0.654	0.513	1
Red–Purple	−74.505	41.427	−1.798	0.072	1
Red–Reference White	89.971	41.427	2.172	0.03	0.627
Red–Yellow	−128.555	41.427	−3.103	0.002	0.04
Blue–Purple	−47.412	41.427	−1.144	0.252	1
Blue–Reference White	62.879	41.427	1.518	0.129	1
Blue–Yellow	101.462	41.427	2.449	0.014	0.301
Purple–Reference White	15.467	41.427	0.373	0.709	1
Purple–Yellow	54.05	41.427	1.305	0.192	1
Reference White–Yellow	−38.583	41.427	−0.931	0.352	1

Note: Each row tests the null hypothesis that the Sample 1 and Sample 2 distributions are the same. Asymptotic significances (2-sided tests) are displayed. The significance level is 0.050. a: Significance values have been adjusted by the Bonferroni correction for multiple tests.

## Appendix G

The Kruskal–Wallis test analysis of people’s responses to colours in VR—logical, lateral and detail abilities.

**Table A13:** Effects on response time (logical thinking abilities).

Pairwise Comparisons of Colours_Logical Thinking Abilities_Response Time_					
Sample 1–Sample 2	Test Statistic	Std. Error	Std. Test Statistic	Sig.	Adj. Sig. a
Green–Blue	3.5	20.601	0.17	0.865	1
Green–Reference White	35	20.601	1.699	0.089	1
Green–Red	38.5	20.601	1.869	0.062	1
Green–Yellow	49	20.601	2.379	0.017	0.365
Green–Orange	−56	20.601	−2.718	0.007	0.138
Green–Purple	−66.5	20.601	−3.228	0.001	0.026
Blue–Reference White	31.5	20.601	1.529	0.126	1
Blue–Red	35	20.601	1.699	0.089	1
Blue–Yellow	45.5	20.601	2.209	0.027	0.571
Blue–Orange	−52.5	20.601	−2.548	0.011	0.227
Blue–Purple	−63	20.601	−3.058	0.002	0.047
Reference White–Red	−3.5	20.601	−0.17	0.865	1
Reference White–Yellow	−14	20.601	−0.68	0.497	1
Reference White–Orange	−21	20.601	−1.019	0.308	1
Reference White–Purple	−31.5	20.601	−1.529	0.126	1
Red–Yellow	−10.5	20.601	−0.51	0.61	1
Red–Orange	−17.5	20.601	−0.849	0.396	1
Red–Purple	−28	20.601	−1.359	0.174	1
Yellow–Orange	−7	20.601	−0.34	0.734	1
Yellow–Purple	−17.5	20.601	−0.849	0.396	1
Orange–Purple	−10.5	20.601	−0.51	0.61	1

Note: Each row tests the null hypothesis that the Sample 1 and Sample 2 distributions are the same. Asymptotic significances (2-sided tests) are displayed. The significance level is 0.050. a: Significance values have been adjusted by the Bonferroni correction for multiple tests.

**Table A14:** Effects on error rate (logical thinking abilities).

Pairwise Comparisons of Colours_Logical Thinking Abilities_Error Rate_					
Sample 1–Sample 2	Test Statistic	Std. Error	Std. Test Statistic	Sig.	Adj. Sig. a
Green–Blue	3.5	20.601	0.17	0.865	1
Green–Reference White	35	20.601	1.699	0.089	1
Green–Red	38.5	20.601	1.869	0.062	1
Green–Yellow	49	20.601	2.379	0.017	0.365
Green–Orange	−56	20.601	−2.718	0.007	0.138
Green–Purple	−66.5	20.601	−3.228	0.001	0.026
Blue–Reference White	31.5	20.601	1.529	0.126	1
Blue–Red	35	20.601	1.699	0.089	1
Blue–Yellow	45.5	20.601	2.209	0.027	0.571
Blue–Orange	−52.5	20.601	−2.548	0.011	0.227
Blue–Purple	−63	20.601	−3.058	0.002	0.047
Reference White–Red	−3.5	20.601	−0.17	0.865	1
Reference White–Yellow	−14	20.601	−0.68	0.497	1
Reference White–Orange	−21	20.601	−1.019	0.308	1
Reference White–Purple	−31.5	20.601	−1.529	0.126	1
Red–Yellow	−10.5	20.601	−0.51	0.61	1
Red–Orange	−17.5	20.601	−0.849	0.396	1
Red–Purple	−28	20.601	−1.359	0.174	1
Yellow–Orange	−7	20.601	−0.34	0.734	1
Yellow–Purple	−17.5	20.601	−0.849	0.396	1
Orange–Purple	−10.5	20.601	−0.51	0.61	1

Note: Each row tests the null hypothesis that the Sample 1 and Sample 2 distributions are the same. Asymptotic significances (2-sided tests) are displayed. The significance level is 0.050. a: Significance values have been adjusted by the Bonferroni correction for multiple tests.

**Table A15:** Effects on response time (lateral thinking abilities).

Pairwise Comparisons of Colours_Lateral Thinking Abilities_Response Time					
Sample 1–Sample 2	Test Statistic	Std. Error	Std. Test Statistic	Sig.	Adj. Sig. a
Red–Orange	−4.2	23.934	−0.175	0.861	1
Red–Green	−6.529	23.934	−0.273	0.785	1
Red–Reference White	42.929	23.934	1.794	0.073	1
Red–Blue	−47.671	23.934	−1.992	0.046	0.974
Red–Purple	−51.543	23.934	−2.154	0.031	0.657
Red–Yellow	−74.029	23.934	−3.093	0.002	0.042
Orange–Green	2.329	23.934	0.097	0.922	1
Orange–Reference White	38.729	23.934	1.618	0.106	1
Orange–Blue	43.471	23.934	1.816	0.069	1
Orange–Purple	−47.343	23.934	−1.978	0.048	1
Orange–Yellow	69.829	23.934	2.918	0.004	0.074
Green–Reference White	36.4	23.934	1.521	0.128	1
Green–Blue	41.143	23.934	1.719	0.086	1
Green–Purple	−45.014	23.934	−1.881	0.06	1
Green–Yellow	67.5	23.934	2.82	0.005	0.101
Reference White–Blue	−4.743	23.934	−0.198	0.843	1
Reference White–Purple	−8.614	23.934	−0.36	0.719	1
Reference White–Yellow	−31.1	23.934	−1.299	0.194	1
Blue–Purple	−3.871	23.934	−0.162	0.871	1
Blue–Yellow	26.357	23.934	1.101	0.271	1
Purple–Yellow	22.486	23.934	0.939	0.347	1

Note: Each row tests the null hypothesis that the Sample 1 and Sample 2 distributions are the same. Asymptotic significances (2-sided tests) are displayed. The significance level is 0.050. a: Significance values have been adjusted by the Bonferroni correction for multiple tests.

**Table A16:** Effects on error rate (logical thinking abilities).

Pairwise Comparisons of Colours_Lateral Thinking Abilities_Error Rate					
Sample 1–Sample 2	Test Statistic	Std. Error	Std. Test Statistic	Sig.	Adj. Sig. a
Green–Reference White	13.971	20.733	0.674	0.5	1
Green–Blue	17.464	20.733	0.842	0.4	1
Green–Orange	−66.364	20.733	−3.201	0.001	0.029
Green–Yellow	68.05	20.733	3.282	0.001	0.022
Green–Red	83.829	20.733	4.043	0	0.001
Green–Purple	−87.321	20.733	−4.212	0	0.001
Reference White–Blue	−3.493	20.733	−0.168	0.866	1
Reference White–Orange	−52.393	20.733	−2.527	0.012	0.242
Reference White–Yellow	−54.079	20.733	−2.608	0.009	0.191
Reference White–Red	−69.857	20.733	−3.369	0.001	0.016
Reference White–Purple	−73.35	20.733	−3.538	0	0.008
Blue–Orange	−48.9	20.733	−2.359	0.018	0.385
Blue–Yellow	50.586	20.733	2.44	0.015	0.309
Blue–Red	66.364	20.733	3.201	0.001	0.029
Blue–-Purple	−69.857	20.733	−3.369	0.001	0.016
Orange–Yellow	1.686	20.733	0.081	0.935	1
Orange–Red	17.464	20.733	0.842	0.4	1
Orange–Purple	−20.957	20.733	−1.011	0.312	1
Yellow–Red	15.779	20.733	0.761	0.447	1
Yellow–Purple	−19.271	20.733	−0.93	0.353	1
Red–Purple	−3.493	20.733	−0.168	0.866	1

Note: Each row tests the null hypothesis that the Sample 1 and Sample 2 distributions are the same. Asymptotic significances (2-sided tests) are displayed. The significance level is 0.050. a: Significance values have been adjusted by the Bonferroni correction for multiple tests.

**Table A17:** Effects on response time (attention to details).

Pairwise Comparisons of Colours Attention to Details_Response Time					
Sample 1–Sample 2	Test Statistic	Std. Error	Std. Test Statistic	Sig.	Adj. Sig. a
Green–Orange	−23.514	23.934	−0.982	0.326	1
Green–Blue	26.143	23.934	1.092	0.275	1
Green–Red	39.343	23.934	1.644	0.1	1
Green–Yellow	59.529	23.934	2.487	0.013	0.27
Green–Purple	−62.129	23.934	−2.596	0.009	0.198
Green–Reference White	68.543	23.934	2.864	0.004	0.088
Orange–Blue	2.629	23.934	0.11	0.913	1
Orange–Red	15.829	23.934	0.661	0.508	1
Orange–Yellow	36.014	23.934	1.505	0.132	1
Orange–Purple	−38.614	23.934	−1.613	0.107	1
Orange–Reference White	45.029	23.934	1.881	0.06	1
Blue–Red	13.2	23.934	0.552	0.581	1
Blue–Yellow	33.386	23.934	1.395	0.163	1
Blue–Purple	−35.986	23.934	−1.504	0.133	1
Blue–Reference White	42.4	23.934	1.772	0.076	1
Red–Yellow	−20.186	23.934	−0.843	0.399	1
Red–Purple	−22.786	23.934	−0.952	0.341	1
Red–Reference White	29.2	23.934	1.22	0.222	1
Yellow–Purple	−2.6	23.934	−0.109	0.913	1
Yellow–Reference White	9.014	23.934	0.377	0.706	1
Purple–Reference White	6.414	23.934	0.268	0.789	1

Note: Each row tests the null hypothesis that the Sample 1 and Sample 2 distributions are the same. Asymptotic significances (2-sided tests) are displayed. The significance level is 0.050. a: Significance values have been adjusted by the Bonferroni correction for multiple tests.

**Table A18:** Effects on error rate (attention to details).

Pairwise Comparisons of Colours Attention to Details_Error Rate					
Sample 1–Sample 2	Test Statistic	Std. Error	Std. Test Statistic	Sig.	Adj. Sig. a
Blue–Reference White	14	20.677	0.677	0.498	1
Blue–Green	−35	20.677	−1.693	0.091	1
Blue–Red	70	20.677	3.385	0.001	0.015
Blue–Yellow	73.5	20.677	3.555	0	0.008
Blue–Orange	−77	20.677	−3.724	0	0.004
Blue–Purple	−87.5	20.677	−4.232	0	0
Reference White–Green	−21	20.677	−1.016	0.31	1
Reference White–Red	−56	20.677	−2.708	0.007	0.142
Reference White–Yellow	−59.5	20.677	−2.878	0.004	0.084
Reference White–Orange	−63	20.677	−3.047	0.002	0.049
Reference White–Purple	−73.5	20.677	−3.555	0	0.008
Green–Red	35	20.677	1.693	0.091	1
Green–Yellow	38.5	20.677	1.862	0.063	1
Green–Orange	−42	20.677	−2.031	0.042	0.887
Green–Purple	−52.5	20.677	−2.539	0.011	0.233
Red–Yellow	−3.5	20.677	−0.169	0.866	1
Red–Orange	−7	20.677	−0.339	0.735	1
Red–Purple	−17.5	20.677	−0.846	0.397	1
Yellow–Orange	−3.5	20.677	−0.169	0.866	1
Yellow–Purple	−14	20.677	−0.677	0.498	1
Orange–Purple	−10.5	20.677	−0.508	0.612	1

Note: Each row tests the null hypothesis that the Sample 1 and Sample 2 distributions are the same. Asymptotic significances (2-sided tests) are displayed. The significance level is 0.050. a: Significance values have been adjusted by the Bonferroni correction for multiple tests.

## Appendix H

The independent *t*-test analysis of people’s cognitive performance between PE and VR.

**Table A19:** The independent *t*-test analysis of people’s cognitive performance between PE and VR.

Independent-Samples Test										
		Levene’s Test for Equality of Variances		T-Test for Equality of Means						
		F	Sig.	t	df	Sig. (2-tailed)	Mean Difference	Std. Error Difference	95% Confidence Interval of the Difference	
									Lower	Upper
Response_Time_Reference_White	Equal variances assumed	0.095	0.758	−1.125	418	0.261	−5.02244	4.46626	−13.80156	3.75668
	Equal variances not assumed			−1.125	404.524	0.261	−5.02244	4.46626	−13.80241	3.75753
Error_Rate_Reference_White	Equal variances assumed	9.726	0.002	1.613	418	0.108	0.07619	0.04724	−0.01667	0.16905
	Equal variances not assumed			1.613	417.317	0.108	0.07619	0.04724	−0.01667	0.16905
Time_Red	Equal variances assumed	3.704	0.055	−1.016	418	0.31	−4.1395	4.07381	−12.1472	3.86821
	Equal variances not assumed			−1.016	371.698	0.31	−4.1395	4.07381	−12.1501	3.87111
Error_Red	Equal variances assumed	0.564	0.453	−1.366	418	0.173	−0.06667	0.04879	−0.16258	0.02925
	Equal variances not assumed			−1.366	417.997	0.173	−0.06667	0.04879	−0.16258	0.02925
Time_Yellow	Equal variances assumed	0.691	0.406	−0.523	418	0.601	−3.05362	5.83804	−14.52919	8.42196
	Equal variances not assumed			−0.523	352.862	0.601	−3.05362	5.83804	−14.53535	8.42811
Error_Yellow	Equal variances assumed	22.21	0	−4.004	418	0	−0.19048	0.04757	−0.28398	−0.09697
	Equal variances not assumed			−4.004	416.798	0	−0.19048	0.04757	−0.28399	−0.09697
Time_Blue	Equal variances assumed	0.393	0.531	0.457	418	0.648	1.96233	4.29829	−6.48664	10.41129
	Equal variances not assumed			0.457	411.295	0.648	1.96233	4.29829	−6.48704	10.41169
Error_Blue	Equal variances assumed	31.096	0	3.148	418	0.002	0.14762	0.04689	0.05544	0.2398
	Equal variances not assumed			3.148	415.472	0.002	0.14762	0.04689	0.05544	0.2398
Time_Green	Equal variances assumed	0.764	0.383	−0.35	418	0.726	−1.28553	3.67239	−8.50418	5.93313
	Equal variances not assumed			−0.35	348.614	0.727	−1.28553	3.67239	−8.50836	5.9373
Error_Green	Equal variances assumed	25.671	0	−2.53	418	0.012	−0.10952	0.04329	−0.19462	−0.02443
	Equal variances not assumed			−2.53	411.417	0.012	−0.10952	0.04329	−0.19462	−0.02443
Time_Orange	Equal variances assumed	8.937	0.003	0.918	418	0.359	3.30378	3.59988	−3.77234	10.3799
	Equal variances not assumed			0.918	395.567	0.359	3.30378	3.59988	−3.7735	10.38106
Error_Orange	Equal variances assumed	0.153	0.696	−2.551	418	0.011	−0.12381	0.04853	−0.21921	−0.02841
	Equal variances not assumed			−2.551	417.998	0.011	−0.12381	0.04853	−0.21921	−0.02841
Time_Purple	Equal variances assumed	8.182	0.004	1.931	418	0.054	9.54971	4.94659	−0.17357	19.273
	Equal variances not assumed			1.931	331.919	0.054	9.54971	4.94659	−0.1809	19.28032
Error_Purple	Equal variances assumed	1.29	0.257	−0.588	418	0.557	−0.02857	0.04862	−0.12415	0.067
	Equal variances not assumed			−0.588	417.985	0.557	−0.02857	0.04862	−0.12415	0.067
